# A Review of Web-Assisted Tobacco Interventions (WATIs)

**DOI:** 10.2196/jmir.989

**Published:** 2008-11-06

**Authors:** Beth C Bock, Amanda L Graham, Jessica A Whiteley, Jacqueline L Stoddard

**Affiliations:** ^3^National Cancer InstituteDivision of Cancer Control and Prevention Science / Behavioral Research ProgramTobacco Control Research BranchRockvilleMDUSA; ^2^Georgetown University Medical Center / Lombardi Comprehensive Cancer CenterWashingtonDCUSA; ^1^The Miriam Hospital Centers for Behavioral and Preventive MedicineWarren Alpert School of Medicine at Brown UniversityProvidenceRIUSA

**Keywords:** Smoking cessation, Internet interventions, tobacco dependence

## Abstract

**Background:**

The Internet has great potential to provide assistance to millions of smokers who seek help with quitting smoking.

**Objective:**

The goals of this study were to assess the content and the quality of smoking cessation treatments most likely to be encountered by smokers seeking treatment on the Internet and to examine differences in quality between current websites and those reviewed in 2004.

**Methods:**

Internet searches for smoking cessation were designed to mimic the search patterns of most Internet users. PhD-level specialists in tobacco cessation treatments used standardized procedures to review the content of each website, assess the degree to which each site covered key components of evidence-based treatment as described in US national guidelines, determine the accuracy of information presented, and evaluate the use of website interactivity. Results of the current study were compared to results obtained in a prior review.

**Results:**

Most websites retrieved in the search met exclusion criteria and were not included in the final analyses in both the current (74%, 65/88) and the prior study (77%, 156/202). In both studies, the majority of websites were excluded because they sold cessation-related products but did not provide treatment recommended by the Public Health Service guidelines. Of the 23 websites included in the current study, 26% (n = 6) provided only minimal coverage (brief mention) of key components of tobacco treatment. However, compared to the earlier study, websites included in the present study scored significantly higher in quality ratings in four areas: providing advice to quit (*P =* .05), practical counseling (*P =* .02), and enhancing motivation to quit smoking through personal relevance (*P* = .05) and risks (*P <* .001). Most Web-assisted tobacco intervention (WATI) sites (69%, 16/23) contained no inaccurate information. When observed, inaccuracies primarily occurred in content related to pharmacotherapy. The percentage of sites offering at least one interactive feature increased from 39% (18/46) in 2004 to 56% (13/23) in the present study. Despite this improvement, there was a notable underutilization of the interactive capabilities of the Internet to personalize treatment, to connect users with a virtual support system, and to provide follow-up treatment contacts.

**Conclusions:**

While the quality of treatment offered in WATIs has improved since our previous review in 2004, there is substantial room for further improvement to ensure that smokers are offered high-quality, evidence-based treatments. It is not clear what degree of informational detail and interactivity is optimal for Web-based smoking cessation treatments. Additional research is needed to understand how to maximize the interactive capabilities of the Internet to produce and sustain population-based health behavior change.

## Introduction

The most recent national data show that 20.9% of US adults are current smokers [1]. Although this is down slightly from the 2001 prevalence of 22%, this rate of decline is not sufficient to meet national health objectives for 2010 [2]. This slow decline, however, should not be interpreted as a lack of interest among smokers in stopping smoking; over 42% of smokers try to quit smoking each year [1]. To impact the population prevalence of smoking, it is critical that smokers be provided with the highest quality, accessible, evidence-based cessation interventions.

The Internet is a widely accessible delivery channel that has great potential to reach millions of smokers with evidence-based treatments. Increasingly, smokers are using the Internet for cessation assistance; recent reports estimate that, annually, over 10 million Americans search the Internet for information and support to quit smoking [3,4]. The Internet is an appealing resource for many smokers who are trying to quit because of its 24/7 “around-the-clock” availability, ease of access, and potential availability of support and encouragement from professional counselors and/or peers.

In response to this demand, there are now hundreds of smoking cessation websites. Several earlier reviews have found that most sites were of mediocre quality [5-7] and that the highest quality websites attract few visitors [7]. In our earlier review, published in 2004, we found that the majority (> 77%) of websites likely to be encountered by smokers searching the Internet did not provide directed guidance or assistance in quitting. We also found that more than 80% of the sites that did provide treatment did not cover one or more of the key components of cessation treatment as recommended by national guidelines [8]. Since our original review, a growing number of randomized trials have been conducted or are currently underway to examine the efficacy of several government, for-profit, and academic cessation websites [9-16]. There has also been increased attention to the development and dissemination of quality standards for health-related websites [17,18]. However, the vast majority of cessation sites remain untested with regard to both efficacy and quality [5-7].

Based on these developments, we were interested in determining whether the landscape of smoking cessation websites had changed since our previous review. Our goals in this study were (1) to assess the content and the quality of Web-assisted tobacco interventions (WATIs) most likely to be encountered by smokers and (2) to determine the extent to which WATIs have changed since our earlier review [5]. Specifically, we were interested in examining whether sites had become more sophisticated in using the interactive capabilities of the Internet and whether the content provided by current WATIs was more consistent with national cessation treatment guidelines [8].

## Methods

### Searches

The first step was to locate WATI websites. Our approach was designed to mimic the search patterns of most Internet users. Several reports have shown that the majority of Internet users tend to use only one search engine and stop at the first page of search returns [19,20]. The most commonly used Web search engines (with number of annual unique visits in parentheses) are Google (89.9 million), Yahoo! (68 million), MSN (49.7 million), Ask Jeeves (43.7 million), and AOL (36.1 million) [20,21]. Therefore, we restricted our review of WATI websites to those that appeared on the first page of a cessation-related search engine query using one of these five search engines. This approach differed from our earlier review, which used a comprehensive search pattern comprising the first 10 pages of search returns obtained using multiple meta-search engines.

Standardized Internet searches were conducted by entering “smoking,” “smoking cessation,” “quit smoking,” and “stop smoking” as the Boolean text string into each of the selected search engines. We compiled a list of all websites retrieved within the first page of search results for each search engine into a list of potential WATI sites for review. Redundant sites returned by more than one search were eliminated from the list, resulting in a final list of 88 unique websites. Using a standardized form, trained coders reviewed the content of each website to determine whether it should be included in this review. Coders reviewed the website’s home page and first level and second level content pages. If relevant content was not detected within these three levels of exploration, the site was excluded from our review. Websites that did not provide direct tobacco treatment services via the Internet were excluded from analysis. For the purposes of this study, “treatment” was operationally defined as the provision of organized, directive information and support services relevant to the process of quitting smoking. Websites were excluded from analysis if they met one or more of the following criteria: (1) product sales only (no treatment components as recommended by the Public Health Service [PHS] guidelines were available on the website itself), (2) libraries (sites that contained articles about smoking, smoking cessation, tobacco policy, advocacy, addiction, or other related topics, but which provided no clear organization or guidance for the smoker who wished to quit), (3) links (sites that only contained links to other sites, including website links and references to hotline phone numbers and bookstores [eg, Amazon.com]), (4) clinics and practitioners advertising face-to-face services, (5) advocacy and political action sites, (6) professional education and information sites designed for health care providers, (7) dead or abandoned websites (eg, a return of “404 file not found” or similar), and/or (8) site content was not smoking-related. The 23 websites reviewed in the current study and the websites reviewed in the 2004 study are presented in Table 1. As is evident from the table, of the 46 sites reviewed in 2004 and the 23 sites included in the present review, 9 were reviewed at both time points.

**Table 1:** Websites reviewed

2004	2007
1. QuitNet.com^a^	1. QuitNet.com^a^
2. SurgeonGeneral.gov/tobacco	2. SurgeonGeneral.gov/tobacco
3. Quitsmoking.about.com	3. Quitsmoking.about.com^a^
4. TryToStop.org	4. TryToStop.org
5. Cancer.org/tobacco	5. Cancer.org/tobacco
6. LungUSA.org^a^	6. LungUSA.org
7. CDC.gov/tobacco	7. CDC.gov/tobacco
8. http://equinox.unr.edu/ homepage/shubinsk	8. http://equinox.unr.edu/ homepage/shubinsk
9. Quit.org.au	9. Quit.org.au
10. stop-tabac.ch^a^	10. Smoking-cessation.org^a^
11. Cancer.ca/tobacco^a^	11. GivingUpSmoking.co.uk^a^
12. ashline.org^a^	12. StopSmokingCenter.net^a^
13. QuitSmokingSupport.com	13. WebMD.com
14. MindFocus.com	14. N/A^b^
15. QuitSmokingUK.com	15. Best-StopSmokingProducts.org
16. Nicotine-Anonymous.org	16. Quit-smoking-guide.com
17. QuitSmokingIn7Days.com	17. RealOvercoming.com
18. Habitrol.com	18. WhyQuit.com
19. DrKoop.com	19. Quit-smoking-review.com
20. QuitSmoking.com	20. SmokeFree.gov
21. nicorette.com	21. Quit.com
22. Zyban.com	22. MostImportantGift.com
23. LifeSign.com	23. SmokingTown.com
24. Smokehelp.org	
25. TobaccoFree.com	
26. HeliosHealth.com/quit_smoking	
27. 123-quit-smoking.com	
28. Smokestoppers.com	
29. LifeClinic.com/focus/smoking	
30. Quit4life.com/html	
31. QuitSmokingHelper.com	
32. HeartScreen.com/smoking_info.html	
33. SmokingHealthLine.com	
34. QuitCommit.com	
35. QuitSmokingOnLine.com	
36. QuitTobacco.org	
37. UCanQuit.com	
38. QuitTobacco.com	
39. WellMD.com/QuitSmokingMD.htm	
40. AHCPR.gov /consumer/helpsmok.htm	
41. MedUMich.edu/1libr/primry/life04.htm	
42. Worldzone.net/health/quitsmoking	
43. InfoTobacco.com	
44. Hoptechno.com/book43.htm	
45. SmokeFreeVirginia.org	
46. MiddlesexHealth.org/health/smoking	

^a^Five highest ranked websites for coverage of key topic areas.

^b^No longer a smoking cessation website as of March 2008.

### STS-C: Assessment of Content

The first assessment instrument used in this study was the Smoking Treatment Scale - Content (STS-C). Details of the development of the STS-C are described elsewhere [5]. In brief, the STS-C is a 12-item checklist on which website reviewers documented the extent to which each website covered material related to key components of treatment as described in the US PHS guidelines for the treatment of tobacco dependence [8]. Key components of the guidelines are codified into operationally defined units and, where appropriate, are subdivided into separate topic areas when the guidelines specified more than one type of action or intervention within the relevant key component. The resulting 12 items on the STS-C are (1-2) advise every smoker to quit smoking (subdivided into two categories: clear/strong and personalized), (3) assess readiness to quit, (4-5) assist with a quit plan (subdivided into three actions related to setting a quit date and seven topics for providing practical counseling), (6) provide intra-treatment social support, (7) recommend use of approved pharmacotherapy, (8) arrange follow-up, and four areas aimed at enhancing motivation to quit by discussing the (9) relevance of quitting smoking, (10) the risks of continued smoking, (11) the rewards of quitting, and (12) the potential roadblocks or barriers to quitting smoking. Reviewers also used the STS-C to document specific examples from each website relevant to the key components being rated.

### STS-R: Rating Website Content

The second assessment instrument used was the Smoking Treatment Scale - Rating (STS-R), which was developed to provide numeric ratings of quality of coverage for each of the key components of treatment documented in the STS-C. Development of the STS-R is described in detail elsewhere [5]. Each website received ratings for (1) coverage, (2) accuracy, and (3) interactivity. Coverage ratings were used to indicate the relative depth and breadth of the information provided in each topic area. Ratings use a 5-point scale. If the treatment component was not mentioned, it received a rating of 1. If the topic was mentioned very briefly, it received a 2. Key components covered briefly but with sufficient detail to be adequately helpful to smokers seeking to quit were given a rating of 3. Sites that provided more detail and more extensive information were given ratings of either 4 or 5 depending on the extent of the information provided. This method is similar to those of Berland et al [22] and was used in our prior review [5]. The overall interrater reliability of the STS-R kappa obtained in the previous study was .77 or greater for all items, ranging from .77 to .93.

Accuracy was rated on a 3-point scale: 3 = “totally correct,” 2 = “mostly correct,” and 1 = “significant misinformation or potentially dangerous errors.” When no inaccurate information was observed, the website received a 3 for that specific component. Where inaccurate information was detected, a rating of 2 was given if the rater judged that the discrepancy was minor and would be unlikely to have harmful effects on site users. In cases where inaccurate information could be potentially dangerous to users (eg, suggesting only palliative remedies for symptoms that could be indicative of nicotine toxicity), a rating of 1 was given.

Reviewers also rated (“yes/no”) whether the website incorporated a user-interactive feature for key treatment components. Interactive features include any content-related user input that results in feedback from the website. Examples of interactive features include entering a target quit date that subsequently generates a quitting calendar or follow-up contact via email; quizzes and assessments that generate individually tailored feedback; chat rooms, bulletin boards, or other interactive community features; interactive recommendations for pharmacotherapy; or the availability of an online pharmacy where medicine could be purchased.

### Procedures

All reviewers were PhD specialists in smoking cessation research and treatment. Reviewers were selected for their clinical or scientific experience, familiarity with the PHS guidelines for the treatment of tobacco dependence, and current research interests or clinical specialization in tobacco dependence treatment. No reviewer had consulted for or had any financial interest or involvement with any of the websites they were assigned to review. Four additional websites (not included in the 23 sites in the analyses) were reviewed for training purposes. After each training review, panel members met to discuss the review process, compare outcomes, and resolve discrepancies. Reviews of each site were conducted independently by two reviewers assigned to each website. Each reviewer used the standardized assessment instruments, which were provided with detailed instructions. Websites were first assessed for content using the STS-C. Results of the content review were used to assign numerical ratings of content quality using the STS-R.

### Analytic Methods

All data were analyzed using SPSS 13.0 statistical software for the PC (SPSS Inc, Chicago, IL, USA). The unit of analysis was the specific URL for all assessments. Interrater reliability was computed for all items on the STS-R. A standard measure of reliability was calculated, computed as the correlation in ratings between reviewers assigned to the same website. Two reviews were included in each calculation of interrater reliability. The overall interrater reliability of the STS-R kappa was .76 or greater for all items, ranging from .76 to .89. Frequency distributions were calculated for each item on both assessment instruments. To examine changes in the quality of WATIs over time, data from the current analyses were compared to the database used in our prior study [5]. Chi-square and analysis of variance (ANOVA) tests were used to assess changes in website quality from our earlier review to the present study.

## Results

### Search Results

Of the original 88 websites returned from the searches, 65 (74%) were excluded from the review. The most common reasons for exclusion were product sales without smoking cessation treatment available directly on the website (66.2% of excluded sites, 43/65), unguided library of articles (21.5%, 14/65), and websites that only provided links to other websites (16.9%, 11/65). Twenty-three percent of websites met more than one exclusion criteria.

### STS-C: Content Coverage

Using the PHS clinical practice guideline [8] as a framework, we examined the degree of coverage for subtopics within each key treatment component area using frequency distributions. Among sites that provided assistance with a quit plan (91%, 21/23), 90% (19/21) encouraged setting a target quit date, 80% (17/21) discussed the notion of planning to quit, and 90% (19/21) discussed making behavioral changes in preparation for quitting. All reviewed websites provided some form of practical counseling; however, there was wide variability in the degree of coverage for each topic within this key component. Nearly all sites (91%, 21/23) included content about the importance of telling family, friends, and/or coworkers about quit attempts and obtaining social support. Less well covered were the following: removing tobacco products from the environment and avoiding alcohol consumption (each 66%, 14/21), the importance of maintaining complete abstinence after quit day (39%, 8/21), and dealing with other smokers in the household (39%, 8/21). Relatively few websites (26%) prompted users to reflect back on lessons learned from prior quit attempts.

While a large majority (87%, 20/23) of websites recommended the use of pharmacotherapy for smoking cessation, two sites warned users against using nicotine replacement therapy (NRT), and one site suggested that while NRT was useful, herbal preparations were preferable (ie, “equally effective with fewer side effects”). Among sites recommending medications, only 55% (11/20) provided explanations of how these medications worked, 45% (9/20) gave instructions on how to use these products, and only 35% (7/20) assessed nicotine dependence. Approximately, one-quarter of websites (6/23) asked users to identify negative consequences of smoking and benefits of quitting that were personally relevant.

### STS-R: Rating Website Content

#### Coverage

Of the 23 websites reviewed that provided smoking cessation treatment, 26% (6/23) did not score above 2 (minimal) for any of the key treatment components. Areas most likely to be covered included “provide practical counseling” (100%), “assist with a quit plan” (91%, 21/23), and “recommend pharmacotherapy” (87%, 20/23). Only 47% of sites (11/23) provided more than adequate or extensive coverage (score of 4 or 5) for any key component. The key components most likely to be given extensive coverage were “assist with a quit plan” (22%, 5/23), “provide practical counseling” (26%, 6/23), and “enhance motivation” (relevance = 22%, 5/23; risks = 26%, 6/23). In contrast, providing clear, strong, and personalized advice to quit (0%) and arranging follow-up contact (4%, 1/23) were least likely to be treated extensively across websites. These results are presented in Table 2.

#### Accuracy

Overall, the accuracy of information provided by most websites was generally high. Reviewers noted no inaccurate information in 69% of websites (16/23). Minor errors were noted in about 30% of websites (7/23) and were most likely to be found for these key components: “assist with a quit plan” (22%, 5/23), “provide practical counseling” (30%, 7/23), and “recommend pharmacotherapy” (35%, 8/23). For example, in the area of “assist with a quit plan,” one site recommended against setting a target quit day or planning ahead. In “provide practical counseling,” some sites provided links to unproven treatments or offered advice that minimized the risk of drinking alcohol while quitting. Inaccurate information regarding pharmacotherapy included recommending hypnosis as “proven to be more than three times more effective than nicotine replacement,” recommending unproven (typically “herbal” or “laser”) remedies, and advising against using nicotine replacement (eg, referring to NRT as a “natural poison” while endorsing herbal remedies). More than 17% of websites (4/23) contained serious or potentially dangerous errors with regard to pharmacotherapy guidance. For example, one website recommended the use of relaxation techniques to reduce symptoms that could indicate nicotine toxicity; this recommendation made no mention of modifying the dosage of NRT or consulting a physician. In assisting with a quit plan, one website advised against making any specific plans to quit and advised that “cold turkey” was the only way to quit.

#### Interactivity

We examined data across all key treatment components, regardless of whether any coverage was provided for that key component, and found that 56% of websites (13/23) provided at least one interactive feature, and 39% (9/23) provided two or more interactive features. Among websites that provided coverage for the relevant key treatment content area, the topics most likely to have interactive features were in the areas of “provide social support” (78%, 15/19), “recommend pharmacotherapy” (45%, 9/20), and “enhance motivation - risks” (47%, 8/17). Only one-third of websites (8/23) used interactive features to assess readiness to quit smoking. The remaining sites asked users to select content based on perceived readiness to quit smoking. Approximately one-third (35%, 8/23) of websites contained links to online pharmacies.

**Table 2:** Website coverage, accuracy, and interactivity for key components of tobacco dependence treatment; percentages of all sites reviewed (n = 23) within each category

	Coverage (Does site cover the essential elements of key topics?)					Accuracy (How accurate is the information?)			Interactive (Is feature Interactive?)
	None	Minimal	Adequate	More Than Adequate	Extensive	Incorrect or Potentially Dangerous	Mostly Correct/Small Errors	Totally Correct/No Errors	Yes
1. Advise every tobacco user to quit: strong	61	17	13	9	0	0	0	100	0
2. Advise every tobacco user to quit: personalized	65	9	9	17	0	0	5	95	14
3. Assess readiness to quit	65	13	9	13	0	0	5	95	15
4. Assist with quit plan	9	35	17	17	22	4	17	78	13
5. Provide practical counseling	0	30	30	13	26	0	30	70	22
6. Provide intra-treatment social support	18	27	27	9	18	0	0	100	40
7. Recommend pharmacotherapy	13	44	22	17	4	17	17	65	30
8. Arrange follow-up	78	4	4	4	9	0	0	100	19
**Enhance Motivation:**									
9. Relevance	22	26	13	17	22	0	0	100	17
10. Risks	26	22	9	17	26	0	5	95	27
11. Rewards	26	13	17	30	13	0	5	95	9
12. Roadblocks	13	30	22	22	13	0	0	100	9

**Table 3:** Differences in mean coverage rating scores of websites between 2004 and 2007 review^a^

	2004 (N = 45), Mean (SD)	2007 (N = 23), Mean (SD)	Difference in Score	*P*
1. Advise every tobacco user to quit: strong	1.41 (.68)	1.70 (1.0)	0.29	.15
2. Advise every tobacco user to quit: personalized	1.33 (.59)	1.78 (1.2)	0.45	.05
3. Assess readiness to quit	1.63 (.77)	1.70 (1.1)	0.07	.48
4. Assist with quit plan	2.74 (.65)	3.09 (1.3)	0.35	.24
5. Provide practical counseling	2.74 (.58)	3.30 (1.1)	0.56	.02
6. Provide intra-treatment social support	2.65 (.58)	2.74 (1.3)	0.09	.49
7. Recommend pharmacotherapy	2.41 (.70)	2.57 (1.0)	0.16	.35
8. Arrange follow-up	1.30 (.71)	1.61 (1.2)	0.31	.74
**Enhance Motivation:**				
9. Relevance	2.30 (.73)	2.91 (1.5)	0.61	.05
10. Risks	2.13 (.71)	2.96 (1.6)	0.83	< .001
11. Rewards	2.41 (.68)	2.91 (1.4)	0.50	.11
12. Roadblocks	2.57 (.69)	2.91 (1.2)	0.34	.29

^a^Rating scale: 1 = None; 2 = Minimal; 3 = Adequate; 4 = More than adequate; 5 = Extensive.

### Changes in Content and Quality Between 2004 and 2007

ANOVA comparing mean scores on website content ratings between the two datasets showed improvements in providing personalized advice to quit smoking (F_1,68_ = 3.82, *P* = .05), providing practical counseling (F_1,68_ = 5.5, *P =* .02), and enhancing motivation through a discussion of the relevance of quitting (F_1,68_ = 3.8, *P* = .05) and the risks of continued smoking (F_1,68_ = 7.1, *P* < .001). These results are shown in Table 3. No comparisons showed any significant decrease in intervention quality between the two reviews. We also examined changes in the percentage of websites providing in-depth coverage (rated 4 “more than adequate” or 5 “extensive”) for key content areas. Compared to 2004, significantly more websites in the present dataset provided in-depth coverage in the areas of “assist with a quit plan” (*χ* ^2^_1_ = 3.9, *P* = .04), “provide practical counseling” (*χ* ^2^_1_ = 6.1, *P* = .01), “arrange follow-up” (*χ* ^2^_1_ = 6.4, *P* = .01), and “enhance motivation” by discussing the risks of continued smoking (*χ* ^2^_1_ = 9.6, *P* < .001) and rewards of quitting (*χ* ^2^_1_ = 5.4, *P* = .02). These data are shown in Table 4.

**Table 4:** Percentage of websites offering “more than adequate” or “extensive” coverage of key topic areas in 2004 and 2007 review

	2004	2007	*χ*^2^_1_	*P*
1. Advise every tobacco user to quit: strong	2	9	1.6	.25
2. Advise every tobacco user to quit: personalized	4	17	3.3	.08
3. Assess readiness to quit	9	13	.32	.67
4. Assist with quit plan	17	39	3.9	.04
5. Provide practical counseling	13	39	6.1	.01
6. Provide intra-treatment social support	15	27	1.2	.30
7. Recommend pharmacotherapy	13	22	.87	.42
8. Arrange follow-up	0	13	6.4	.01
Enhance Motivation:				
9. Relevance	15	39	4.9	.07
10. Risks	11	44	9.6	< .001
11. Rewards	17	44	5.4	.02
12. Roadblocks	17	35	2.6	.19

## Discussion

The goal of this study was to examine the content and quality of WATIs that are most likely to be encountered by smokers looking for online cessation assistance. We were also interested to see whether there were changes in quality between websites reviewed in the current study and those of our previous review, published in 2004 [5]. Both studies used standardized procedures and assessment instruments when evaluating websites, and, in general, findings were similar for both reviews. For example, the percentage of websites meeting exclusion criteria was very similar between studies (77%, 156/202 in 2004 and 74%, 65/88 in 2007). In both cases, the most common reasons for exclusion were sites offering only product sales and undirected libraries of articles about smoking, smoking cessation, tobacco advocacy, and other tobacco-related topics. Results from both studies indicate that individuals searching for help with quitting smoking are most likely to encounter websites that do not offer smoking cessation treatment. Web-based interventions should include a clear organizational structure that actively guides users through the treatment process.

Results of this study indicate that while the majority of reviewed websites provide coverage for most key content areas identified as the core of smoking cessation treatment [8], the depth of coverage for key topics was most often minimal. While there are no empirical studies demonstrating how much detail is needed to help smokers quit, it is likely that providing more than a brief mention of important content areas would result in better treatment outcomes. It may be particularly useful for websites to be designed in such a way as to allow users to drill down to their desired level of detail on any given topic. Sites that provide only minimal coverage of important information may do a disservice to smokers who are seeking to quit and need additional information to enhance the quit attempt. However, it is also likely that websites that present too much detailed information on each page may result in users missing important content. Thus, it is not sufficient to consider only general standards of usability [23,24] when designing a behavior change website; it is also critical to understand the ways in which individuals use websites to make behavioral changes (ie, behavioral informatics). For example, some users may prefer to read science-based resources such as quitting guides or published manuscripts, while others may prefer to connect with other smokers in a community forum. Some may feel comfortable using interactive features that yield individually tailored information, while others may have concerns about privacy. Understanding the ways in which users interact with a cessation website and the relative contributions of various treatment components will help advance the science of Web-based behavior change.

Providing practical counseling was the key content area that received the most coverage: all websites provided at least minimal coverage of this topic. This is not surprising given that providing practical counseling and information could be described as the core content area of tobacco dependence treatment. However, within this topic, few websites prompted users to reflect back on prior quit attempts. Such reflection is important as it helps smokers to identify triggers, situations that are high risk for relapse, and techniques they found useful and could employ again [8]. Likewise, while most sites recommended the use of pharmacotherapy, most often NRT, the information provided tended to be superficial and was limited by a lack of explanation regarding instructions for use, contraindications, and potential side effects. This problem was noted in our earlier study and has been discussed in other reviews [6,7]. Content relevant to medication use was also the most likely of all content areas to contain serious errors. In some cases, the error was implicit in that NRT was included in a list of other unproven or unsafe alternatives, which lends a halo of legitimacy to those alternative treatments while also reducing the relative strength of the recommendation for established efficacious treatments such as NRT. In other cases, errors were more explicit, such as stating that using NRT is dangerous and should be avoided.

There was a notable lack in using the capacity of the Internet for personalization of treatment. An important part of motivating smokers to quit is to personalize information relevant to quitting. Reasons for quitting, perceived risks of continued smoking, as well as perceived benefits and barriers to quitting should all be identified by the individual to have maximal impact [25]. The PHS guideline specifies that smoking cessation interventions should encourage smokers to discuss their reasons for wanting to quit and should provide personalized information about the risks of smoking and rewards of quitting. While the majority of websites provided information about the risks and rewards of quitting smoking, these key components were usually treated only with generic lists of benefits and health risks rather than personally relevant messages as specified by the PHS guideline. Approximately one-quarter of websites asked users to identify consequences of tobacco use and benefits of quitting that were personally relevant. Few websites took advantage of the Internet’s unique ability to provide individually tailored feedback. In the current study, benefits of quitting and risks of continued smoking were most often presented as generic lists, with no attempt at personalization.

Related to this issue, the interactive capability of WATIs was generally underutilized. Across all of the key treatment components, only a minority of websites provided interactive features. The most common use of interactivity was in the area of providing intra-treatment social support, frequently in the form of chat rooms, buddy lists, and emailed support. Recommendations for pharmacotherapy were frequently interactive in nature, although limited to the administration and scoring of assessments of nicotine dependence. Perhaps the most glaring failure to leverage the capabilities of the Internet was in providing follow-up contact. Follow-up contacts can be used to motivate smokers to make a quit attempt or to reconsider cessation following slip/relapse and to provide support during difficult times while quitting [8]. Providing follow-up was one of the least used key treatment components observed in WATI sites. In the present study, just over one-fifth of websites provided any sort of treatment follow-up.

Compared with our earlier review, the current results indicate modest improvement in the quality of coverage in key content areas. Areas showing the most improvement were giving personalized advice to quit, providing practical counseling, recommending medications to aid quitting, and enhancing motivation (discussing personal relevance of quitting, perceived risks, and roadblocks to quitting). In no case did we observe a significant decline in the quality of website content. It is encouraging that the quality of some content areas may be improving. However, there remains substantial room for further improvements. Most often, the reviewed websites provided only minimal coverage of key component areas. Only in discussions of risks and roadblocks did most websites provide more than adequate or extensive coverage, and even in these areas, few websites took advantage of the interactive capacity of the Internet to truly personalize treatment.

### Limitations

Results of this study should be considered in the context of several limitations. First, this review should not be considered an exhaustive analysis. In the present study, we reviewed only English-language websites. Thus, the quality of websites available in other languages remains unknown. Given that much of the world is non-English-speaking, we encourage researchers with fluency in other languages to conduct similar reviews of non-English websites. Second, comparisons between the 2004 and current review are made with a notable caveat. The search procedure used in the 2004 paper was comprehensive, including all websites retrieved in the first 10 pages of search returns. The current review used a search strategy that was designed to mimic the search pattern of most Internet users. That is, we included only those websites retrieved on the first page of search returns. It is possible that higher quality websites are more likely to be retrieved in the first page of search returns. If true, the increases in quality observed between the 2004 and current dataset may be an artifact of the search procedures rather than a reflection of a real improvement in the quality of smoking cessation websites. In a review of popular smoking cessation websites identified by survey respondents, Etter [7] concluded that users had difficulty finding the highest quality websites: the three highest rated websites in that review attracted only 7% of visitors. The sites that were most commonly used (ie, the most popular) were not the highest quality. Third, the present study was designed to address the quality of content presented in the websites. Elements of usability such as navigation, layout, and accessibility are also important to a user’s experience and likely play an important role in the effectiveness of a behavior change website. These elements should be examined in future studies.

### Conclusions

Results of this study indicate that the content and quality of information contained in smoking cessation websites may be improving. However, more often that not, smokers looking for assistance online will find websites that do not provide evidence-based guidance and assistance. Moreover, numerous questions remain to be answered about WATIs. Research needs to move beyond quantitative assessments of the amount and accuracy of information provided via the Web and begin to examine the qualitative nature of WATI sites and the relationships that exist between these sites and their users. For example, research is needed to determine whether there are intrapersonal (eg, age, education, health literacy, need/preference for social support) or environmental characteristics (eg, support/incentives from a health maintenance organization or employer, presence of smoking policy restrictions) that predict better or worse outcomes when using Internet-delivered cessation interventions. The Internet holds great potential to reach millions of smokers who may not otherwise seek cessation treatment. Efforts are needed to ensure that the content of Internet interventions is sound so that we can begin to understand how, for whom, and by what mechanism(s) WATIs may be effective.

## Abbreviations

NRT: nicotine replacement therapy
PHS: Public Health Service
STS-C: Smoking Treatment Scale - Content
STS-R: Smoking Treatment Scale - Rating
WATI: Web-assisted tobacco intervention
